# Overdispersed logistic regression for SAGE: Modelling multiple groups and covariates

**DOI:** 10.1186/1471-2105-5-144

**Published:** 2004-10-06

**Authors:** Keith A Baggerly, Li Deng, Jeffrey S Morris, C Marcelo Aldaz

**Affiliations:** 1 Department of Biostatistics and Applied Mathematics, UT M. D. Anderson Cancer Center, Houston, TX, USA; 2 Department of Statistics, Rice University, Houston, TX, USA; 3 Department of Carcinogenesis, UT M. D. Anderson Cancer Center, Houston, TX, USA

## Abstract

**Background:**

Two major identifiable sources of variation in data derived from the Serial Analysis of Gene Expression (SAGE) are within-library sampling variability and between-library heterogeneity within a group. Most published methods for identifying differential expression focus on just the sampling variability. In recent work, the problem of assessing differential expression between two groups of SAGE libraries has been addressed by introducing a beta-binomial hierarchical model that explicitly deals with both of the above sources of variation. This model leads to a test statistic analogous to a weighted two-sample *t*-test. When the number of groups involved is more than two, however, a more general approach is needed.

**Results:**

We describe how logistic regression with overdispersion supplies this generalization, carrying with it the framework for incorporating other covariates into the model as a byproduct. This approach has the advantage that logistic regression routines are available in several common statistical packages.

**Conclusions:**

The described method provides an easily implemented tool for analyzing SAGE data that correctly handles multiple types of variation and allows for more flexible modelling.

## Background

### The nature of SAGE

The Serial Analysis of Gene Expression (SAGE) methodology introduced by Velculescu et al. [1] is a sequencing-based approach to the measurement of gene expression.

Briefly, mRNA transcripts are converted to cDNA and then processed so as to isolate a specific subsequence; starting from the poly-A tail, the subsequence is the 10 (normal SAGE) or 14 (long SAGE) bp immediately preceding the first occurrence of a cleavage site for a common restriction enzyme. Ideally, this subsequence, or "tag" is sufficiently specific to uniquely identify the mRNA from which it was derived. Tags are sampled, concatenated and sequenced, and a table consisting of the tag sequences and their frequency of occurrence is assembled. The complete table derived from a given biological sample is referred to as a SAGE "library". As most tags are sparse within the entire sample, most libraries contain numbers of tags in the tens of thousands to allow the expression levels to be estimated. Due to the current costs of sequencing, however, the total number of libraries assembled for a given experiment is typically small: often in the single digits and occasionally in the tens.

While the type of information, gene expression, being investigated in a SAGE experiment is the same as that in a cDNA or oligonucleotide microarray experiment, there are some qualitative differences in the approaches. First, SAGE uses sequencing as opposed to competitive hybridization. Second, while the expression value reported for an array experiment is a measure of fluourescence and is loosely continuous, SAGE supplies data on gene expression in the form of counts, potentially allowing for a different type of "quantitative" comparison. Third, SAGE is an "open" technology in that it can provide information about all of the genes in the sample. Microarrays, by contrast, are "closed" in that we will only get information about the genes that have been printed on the array.

Mathematically, the information pertaining to the abundance of a particular tag in a sample is summarized in two numbers: *Y*, the number of counts of that tag in the library, and *n*, the total number of tags in the library. In analyzing SAGE data across a series of libraries, interest typically centers on assessing how the underlying true level of gene expression is changing as we move from one library to the next.

### Mathematical formulation of the differential expression problem

When surveyed across a series of libraries, the sufficient statistics containing all of the information about the change in expression for a single tag are the set of counts {*Y*_*i*_} and the set of library sizes {*n*_*i*_}, where the subscript *i *denotes the specific library. Unless otherwise specified, we will restrict our assessment of differential expression to the case of a single tag. This approach is common to all of the procedures described below. In a real analysis the chosen test is applied to all tags individually and a list of those tags showing differential expression is reported. Different tests will provide altered assessments of significance for individual tags, and hence the list provided will depend on the test employed.

In most problems of interest, there is also covariate information *X*_*i *_describing properties of library *i*. The most common case involves comparing two groups of libraries, such as cancer and control. In this case the information *X*_*i *_simply defines which group library *i *belongs to. If there are more than two groups, *X*_*i *_can have more levels or can even be vector valued, but as before interest centers on assessing how and whether the expected proportion changes with *X*.

Much work has been done on the problem of comparing expression between two groups. Most of the approaches [2-9] deal with comparing one library with another. Of these, [2,6,7] extend their consideration to the case of two groups of libraries by pooling the libraries within a group, effectively reducing the sufficient statistics to the summed counts


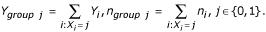


This approach, while it captures the count nature of the data, loses information in that variation of the proportions within a group is ignored. As noted by both Man et al. [9] and Ruijter et al. [10], most of the above tests give equivalent results in terms of assessing significant differences. By contrast, the two-sample *t*-test used to compare two groups of samples in [11] reduces the sufficient statistics to the set of proportions {*p*_*i*_} = {*Y*_*i*_/*n*_*i*_}, capturing the variation between members of a group but losing track of the inherent count sampling nature and variability of the data. The two-sample *t*-test results can be dramatically different from the pooled test results, as they focus on two different types of variation. The effects of these two approaches on a single group of four libraries are shown in Table 1. Pooling reduces the data to the summed counts at the right, and focusing on proportions reduces the data to the proportions on the bottom. In both cases, this reduction results in a loss of information. When pooling is used, we can't tell that one of the group proportions was large and the other small, indicating instability. When proportions are used, we can't tell that one library was much smaller than the other, so that proportion should be "trusted less" than the other.

Baggerly et al. [12] proposed a beta-binomial hierarchical model for SAGE data in an attempt to simultaneously model both types of variation. This model leads to a test statistic called a weighted two-sample *t*-test, *t*_*w*_. Computing the value of this test statistic requires all 8 of the numbers in the main body of Table 1; there is no reduction of the sufficient statistics. This test statistic exhibits different behaviors depending on which type of variation is larger for a given tag. When the within-library sampling variation is much larger than the between-library variation, *t*_*w *_gives results close to those supplied by pooling tests, which focus on within-library variation. Conversely, when the between-library variation is much larger than the within-library variation, *t*_*w *_gives results very similar to those of a two-sample t-test, which focuses on between-library variation. The *t*_*w *_model also allows the relative contributions of the two types of variation to be assessed. Baggerly et al. [12] found that for high-count tags, between-library heterogeneity is the much larger source of variation and that pooling methods which do not allow for heterogeneity are biased towards finding high count tags to be significantly different. This can potentially lead to large fractions of false positives, as becomes apparent when the results for several different tags are plotted.

### Extensions to multiple groups

While cases with more than two groups have been described in the literature [2,13-15], the means of analysis is currently something of a hybrid. Methods explicitly attacking the multi-library problem have been proposed [16,17], but the most common approach at present [13,15] seems to involve coupling hierarchical clustering of the data with pairwise tests for differential expression [2] between one group and another. This hybrid approach can indirectly capture both types of variability, with the hierarchical clustering focused on the variation between proportions within a group, and the pairwise test focusing on sampling variation. Clustering has other benefits for clarifying thought apart from assessing differential expression, and we definitely recommend it for exploring the structure of the data. However, clustering tends not to provide a numerical summary, so combining the clustering results with those of the pairwise comparisons can be something of an art. An additional drawback is that the pairwise comparisons may miss useful information about variability by focusing only on a subset of the libraries available. For the purposes of assessing differential expression we believe more efficient tests are available.

### Our approach: Overdispersed logistic regression

We seek to construct a method that takes the count nature of the data into account, deals with multiple groups simultaneously, and allows for variability in the proportions beyond that due to sampling alone. Fortunately, this is not the first time such a problem has arisen.

The problem of assessing differential expression for multiple groups corresponds to the classical statistical problem of the analysis of variance (ANOVA). When the values of interest are continuous (e.g., microarray log ratios), the test statistics become F-tests, higher-dimensional generalizations of the two-sample *t*-test. When the data are counts (SAGE data), and sampling variability needs to be dealt with, the ANOVA test can be adapted to give logistic or Poisson ANOVA. The multi-library test for differential expression proposed by Stekel et al. [17] corresponds to Poisson ANOVA, but without allowance for overdispersion. ANOVA deals with the extension from two to a larger number of distinct groups, but this can be viewed as a special case of the situation where the covariate information is continuous. One common way of modelling the dependence of proportions upon covariates is through logistic or Poisson regression, both of which are special cases of generalized linear models [18,19]. Such models incorporate the form of the sampling variability directly. For example, the logistic model for proportions,


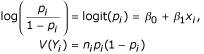


defines both the function of the data that is to be modeled in terms of the covariates (the logit of the proportions) and the precision of each of the measurements. The maximum likelihood estimates of the parameters of this model can be found through iteratively reweighted least squares (IRLS).

Excess variation within a level, or overdispersion, can be introduced into a logistic regression framework in a number of ways. The most common and most widely implemented approach is to replace the binomial likelihood function being maximized above with a "quasi-likelihood" which differs from the initial formulation solely through the introduction of a scale term, 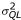, into the variance equation, so that

*V*(*Y*_*i*_) = *n*_*i*_*p*_*i*_(1 - *p*_*i*_)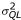.

This approach has the advantage that it inflates the variance of each of the observations by a like amount, so that the estimated 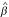 values will be the same – just the associated standard errors will be inflated. Logistic regression with quasi-likelihood overdispersion is implemented in a wide variety of statistical packages, including S-PLUS, R, GLIM, and SAS. Another method of introducing overdispersion is to assume a hierarchical model in which the proportions at a given level of the covariate are drawn from a nondegenerate distribution, and the distribution of the observed counts is binomial conditional on the value of the drawn proportion. When a beta distribution is assumed for the proportions, the final unconditional distribution of the observed counts is beta-binomial. This is the model suggested by Baggerly et al. [12] for modelling overdispersion in SAGE data, and is also the model used by Crowder [20] in generalizing ANOVA to deal with proportions subject to overdispersion. It can be shown (eg, Collett [18] p.201) that the variance of beta-binomial counts is of the form

*V*(*Y*_*i*_) = *n*_*i*_*p*_*i*_(1 - *p*_*i*_)[1 + (*n*_*i *_- 1)*φ*],

which is equivalent to the quasi-likelihood formulation when all of the library sizes *n*_*i *_are the same. While approximate equality may suffice, even this assumption may be questionable for SAGE data, particularly if some of the libraries are drawn from experiments conducted at different times. Williams [21] shows how IRLS can be adapted to deal with this type of overdispersion, and notes that estimation involves *φ *only and need not assume further structure from the beta distribution, making the procedure slightly more general. This form of overdispersion is implemented in R as part of the dispmod package.

In the logistic regression framework, assessing differential expression reduces to a case of deciding whether a set of regression coefficients is different from zero. This can lead to slightly different inferences than models such as *t*-statistics applied to the proportions, in that approximate normality is assumed to hold for the *β *values rather than the proportions themselves. When we have worked with a beta model for the *p*_*i*_'s, we have been led to choices of parameters which yield quite skewed distributions, suggesting that the logit scale may be more appropriate. Working with the *β *values has the additional advantage that confidence intervals are naturally interpreted in terms of fold changes.

## Results

### Comparing two groups

We begin by comparing the counts of the tag ATTTGAGAAG in 8 colon libraries initially described in Zhang et al. [2]. These 8 libraries include two normal colon (NC1 and NC2), two primary tumors (TU98 and TU102) and four cell lines (CACO2, HCT116, RKO, and SW837). For now, we focus on comparing normal colon with all tumors, primary or cell line. Counts of the tag and the corresponding library sizes are given in Table 2. A χ^2 ^test applied to the pooled counts from each of the two groups yields a test statistic of 444.27; the 95% cutoff for the null 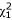 distribution is 3.84, with values above this being deemed "significant". The two-sample *t*-test applied to the two groups of proportions yields 1.60; the 95% cutoffs for the null *t*_6 _distribution are ± 2.45, so this test suggests that the difference is not significant, showing the possibility of stark disagreements between tests focusing on different portions of the variability. The *t*_*w *_statistic proposed by Baggerly et al. [12], which incorporates both types of variance, yields a test statistic of 1.60. The null distribution of this test statistic in this case is approximately a *t*_6 _distribution, and the qualitative results are far closer to those of the *t*-test than those of the pooled tests, reflecting the relative dominance of patient heterogeneity in driving the total variation for this tag. We note in passing that this disagreement between the two types of tests is not an isolated incident. When we surveyed all of the tags in this group of libraries we found 10 tags with |*t*| < 2 and χ^2 ^> 200, and 48 tags with |*t*| < 2 and χ^2 ^> 50. In Baggerly et al. [12] it was found that most high-count tags appeared significantly different when a pooled test was used and not significant when a *t*-test was tried, and that in this case the *t*-test was more likely correct.

The results from three logistic regression model fits to the data are shown in Table 3. In the first model there is no allowance for overdispersion, in the second the quasi-likelihood approach to overdispersion is employed, and in the third the hierarchical approach to overdispersion is used. Here, the values of the covariate *X *are 0 or 1 as the library is in the first or second group, respectively. In models 1 and 2, the fitted proportions are 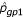 = *e*^-4.66^/(l + *e*^-4.66^) = 0.94% and 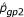 = *e*^-4.66 - 0.89^/(l + *e*^-4.66 - 0.89^) = 0.39% for the first and second groups, respectively, and the proportions are only slightly altered in model 3. We note that the estimated coefficient values are exactly the same for the first two models, and this is true for these two approaches in general. Fitting the model with no allowance for overdispersion gives a *z*-value of *β*_1_/*s.e. *(*β*_1_) = -20.42, which is definitely significant. Note that the square of this value is of the same order as the value found by the χ^2 ^test. The Pearson residuals from this model, 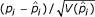, however, show a problem. If the model fits well, these should be approximately distributed as a standard normal, with extreme values from a set of 8 observations around 3 or 4 in magnitude. The actual values, -14.6 and 19.0, are far too extreme. When the model is fit with allowance made for overdispersion, the point estimate of the dispersion parameter is 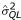 = 187.57; this value should be close to 1 if there is no overdispersion. With this allowance made the *t*-value of -1.49 is no longer significant. This *t*-value can be found from the first *z*-value (-20.42) by dividing by 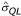 = 13.70. Similarly scaling the residuals yields values far more commensurate with a standard normal. We note that due to the differences in the models employed, the presumed distributions of the test statistics have changed. If we assume that the standard logistic model with no overdispersion holds, the test statistic has an approximately normal distribution. This is because the number of total successes is driving the binomial distributions to approximate normality. When we shift to a model where we presume the existence of overdispersion, the test statistic now has a *t *distribution. This is because our estimate of the variance is now strongly dependent on the precision with which we can estimate the overdispersion parameter, and this precision depends on the number of libraries, not the number of successes. Fitting this model with the hierarchical type of overdispersion, model 3 in Table 3, yields slightly different answers but the size of 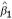 is still not significant. The difference in 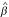 values from those found before is due to the fact that in this model the amount of overdispersion attributed to each proportion changes slightly with library size, thus altering the weights used in the regression model. The point estimate for the hierarchical dispersion parameter *φ *is 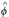 = 3.399*e *- 03, so the multipliers for the binomial variances are

1 + ({*n*_*i*_} - 1)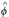 =

(169.62, 165.78, 141.62, 190.32,

207.26, 190.12, 175.35, 208.84).

averaging these gives 181.11 which is close to the value found for the quasi-likelihood dispersion parameter. We note that the differences in coefficient values for models 2 and 3 are largely cosmetic, but the differences in significance between model 1 and the others are not. Choosing to account for overdispersion is more important than the precise model used to achieve this.

We note that the overdispersed logistic regression approaches give *t*-values about -1.49, whereas the two-sample *t*-test and the modified version *t*_*w *_suggested by Baggerly et al. [12] both give *t*-values of about -1.6 (as noted earlier, agreement between *t *and *t*_*w *_suggests that for this tag, the between-library variation is much larger than the within-library variation). There are two reasons for this difference. First, the *t *statistic works on the proportion scale, and logistic regression works on the *β *scale, which is roughly the log proportion scale. Second, the *t*_*w *_statistic used here,


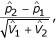


does not assume that the overdispersion factor is the same in the two groups being compared; the variance estimate is not pooled. The latter difference is actually the more important for this contrast, particularly as the variance estimate from the first group of size 2 is very unstable. This effect is not always subtle; if we consider instead the tag GCGAAACCCT, with counts given in Table 2, the two-sample *t *test and the weighted *t*_*w *_test both give -1.57, and the logistic regression *t *value is -4.16.

Of the two answers, we tend to prefer the one given by the logistic regression fit, for two reasons. First, when we have fit the parameters of the beta distributions for the proportions directly, we have found the distributions to be quite skewed. As such we find it better to assume rough normality on the *β *coefficient scale. Second, when the number of libraries in a group is quite small, which will often be the case with SAGE data, we prefer the pooled estimate of the variance. This preference is due in large part to its greater stability through the use of more degrees of freedom. It is possible to explicitly incorporate levels of overdispersion that change with the covariates in logistic regression, but we have not pursued this here.

### Comparing three or more groups

Above, we treated the colon libraries as if they came from two groups, but it is more natural to view them as coming from three: normal samples, primary tumors, and cell lines. When we have data from multiple groups, there are two different ways in which this changes the nature of the problem. First, if we are only interested in comparing two of the groups, it is often nonetheless worthwhile to incorporate the data from the other groups into the model. The reason for this is that when overdispersion is driving the variance, the significance of our results depends strongly on the precision with which we can estimate the overdispersion parameter. The libraries in the groups not directly involved in the comparison of interest can still supply information about the overdispersion parameter and increase the degrees of freedom of the associated t-test. Second, by examining the fitted proportions for all groups, the relative sizes of the transitions can be assessed.

We begin by looking at the results for a single tag flagged as interesting in the paper by Zhang et al. [2], namely TGCTGCCTGT, where we presume that the contrast of most interest is between normal colon and primary tumors. The counts for this tag and the corresponding library sizes are given in Table 2.

We first attempt to compare the levels in normal colon and primary tumor while ignoring the cell lines (ie, using just four libraries), and then using a model incorporating all three groups. The results using logistic regression with hierarchical overdispersion are shown in Table 4.

In the model with only two groups, we have a single covariate vector *x*_1 _= (0,0,1,1) denoting which of the two groups the library belongs to. This model produces an overdispersion estimate of 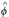 = 8.938*e *- 05, for inflation factors of

1 + ({*n*_*i*_} - 1)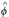 = (5.43, 5.33, 4.70, 5.98).

The fact that these factors are significantly larger than one suggests that the within-group heterogeneity is the dominant component of the variance not explained by the model. In the model with three groups, we cannot use a single covariate vector *x*_1_, as this is not suited to indicating 3 or more groups in an unordered fashion (using 0, 1, and 2 for the three groups respectively would force an ordering by saying that primary tumors are intermediate betwixt normal samples and cell lines). In general, if we have *k *groups, we need to use *k *- 1 covariate vectors. Here, we use *x*_1 _= (0, 0, 1, 1, 0, 0, 0, 0) and *x*_2 _= (0, 0, 0, 0, 1, 1, 1, 1). The set of all 0s (*x*_1 _= 0, *x*_2 _= 0) corresponds to the first group, here normal colon, and the other groups are defined by which one of the other covariates is nonzero: Group 2 (primaries), (*x*_1 _= 1, *x*_2 _= 0), Group 3 (cell lines), (*x*_1 _= 0, *x*_2 _= 1) As we are still focused on the difference between normal colon and primary tumors, for which the logit values are *β*_0 _and *β*_0 _+ *β*_1 _respectively, the main interest remains on whether *β*_1 _is significantly different from zero, and the predicted logit for the cell line group, *β*_0 _+ *β*_2_, does not enter the problem directly. Fitting this model produces an overdispersion estimate of 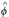 = 1.160*e *- 04, for inflation factors of

1 + ({*n*_*i*_} - 1)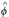 =

(6.76, 6.62, 5.80, 7.46,

8.04, 7.45, 6.95, 8.09).

In neither case (considering two groups or three) does the contrast between normal colon and primary tumor, represented as the magnitude of 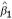, appear significant once allowance is made for overdispersion, but there is an interesting point to note. Even though the point estimate of overdispersion increases when the cell lines are included, and the value of the *t*-statistic (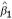/*s.e*(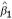)) associated with the difference declines, the associated p-value indicates an increase in significance. Without using the cell lines, we have just 4 libraries, and after estimating the mean proportions in each group just 2 degrees of freedom for estimating *φ*. When we use the cell lines, we have 8 libraries and 5 degrees of freedom for estimating *φ*. Thus, the degrees of freedom in the *t*-tests shift from 2 to 5. The *t*_2 _distribution has very wide cutoffs, and the *t*_5 _is much closer to normal. In general, the inclusion of related groups can improve estimation by increasing the precision of our estimate of overdispersion.

In fitting the model with three groups, of course, we have also gained the ability to look at other contrasts. For example, we can look at normal colon versus cell lines, for which the logits are *β*_0 _and *β*_0 _+ *β*_2 _respectively, by checking the significance of 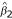. Likewise, we can look at the difference between primary tumors and cell lines, for which the logits are *β*_0 _+ *β*_1 _and *β*_0 _+ *β*_2 _,by testing the significance of the difference 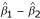. While this significance is not listed in the table directly, we can compute the standard error of this contrast, *s.e. *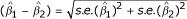, divide the estimate by its standard error to get a t-statistic with the degrees of freedom listed (here 5), and compute a p-value accordingly.

It is also possible to perform an omnibus test of whether there exists any significant difference among the groups, which is logistic ANOVA for proportions. The regular ANOVA test looks at the amount of variance explained by the terms of interest in the model and compares this to the amount of residual variance. Adjusting for the degrees of freedom in each group gives an *F*-test. When dealing with generalized linear models, the quantity -2 * log (likelihood ratio), known as the deviance, plays a role analogous to the variance in ANOVA and thus we can speak of the analysis of deviance. The analysis of deviance is complicated by the inclusion of overdispersion in the model, requiring a multi-step approach in which several different models are fit in succession. These models are listed in Table 5. First, the model is fit using all available covariates and the overdispersion parameter is estimated. Here, the available covariates are *x*_1 _and *x*_2_, and fitting the full model with both present, *β*_0 _+ *β*_1 _+ *β*_2_, gives 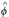 = 1.160*e *- 04 as noted above. Second, submodels are fit with the value for overdispersion fed in as fixed. In this case, the submodels are *β*_0 _+ *β*_2_, using *x*_2 _as the only covariate, *β*_0 _+ *β*_1_, using *x*_1 _as the only covariate, and *β*_0_, using no covariates and simply fitting a single proportion to all of the data. The results are shown in Table 5, from which the significance of a given model can be assessed by comparing the scaled reduction in deviance with the scaled residual deviance to the appropriate *F *distribution. Here, for example, testing whether the overall model including *β*_1 _and *β*_2 _explains things significantly better than just fitting the same proportion throughout (*β*_0_) reduces to


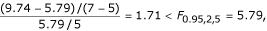


indicating that the overall difference between groups is not significant at the 5% level. It may be noted that the submodel including just *β*_1 _in addition to the constant appears to explain very little; this is due to the way in which we have chosen the entries of *X*, so that including *β*_1 _isolates the effect of the primary tumor group, but excluding *β*_2 _still combines the normal colon group with the cell line group for the contrast. This latter grouping blurs the normal colon vs primary tumor distinction found to be a bit larger earlier.

### Incorporating other covariates

It is possible to use the logistic regression approach to partition the variance amongst multiple effects of interest. For example, in the above section we considered a case with colon libraries taken from both primary tumors and cell lines. Such data is also available for other organs, eg pancreas. If we are interested in identifying consistent differences between primary tumors and cell lines, it would be natural to use libraries from both organ types. However, if these were then compared as two groups, primary vs cell lines, the differences would be difficult to isolate due to the large differences between tissue types within both the primary and cell line groups. The solution is to fit a model with two covariates, with *x*_1 _being 0 or 1 as the sample is colon or pancreatic, respectively, and *x*_2 _being 0 or 1 as the sample is a primary tumor or a cell line, respectively. Inference reduces to testing the significance of *β*_2_, with the scale of natural variation being assessed only after the effects of the change in tissue type, *β*_1_, have been factored out.

In the above example, we allowed for the effect of one other effect, tissue type. In principle, multiple factors can be allowed for through the inclusion of other covariates. Likewise, though the two covariates in the above example were both "factors" having a finite number of unordered levels, it is possible to include continuous covariates in the modelling process as well.

To illustrate this, we give two hypothetical examples using the counts for the GCGAAACCCT tag from Table 2. In the first example, we posit that we are trying to assess the differences between normal tissue and primary tumors, that the first 4 libraries come from normal colon and primary tumors as indicated, and that the remaining 4 libraries come not from cell lines but rather from normal tissue (libraries 5 and 6) and primary tumor (libraries 7 and 8) from some other organ. As noted above, this leads to a scenario where we want to fit a model with two covariates: *x*_1 _= (0, 0, 1, 1, 0, 0, 1, 1), indicating whether the library is normal (0) or primary tumor (1), and *x*_2 _= (0, 0, 0, 0, 1, 1, 1, 1), indicating the organ from which the library was derived. In the second example, we posit that in addition to the above information, we have access to the levels of a biomarker potentially predictive of survival. These levels are supplied as the values of a third covariate vector, *x*_3 _= (0.89, 0.35, 0.66, 0.23, 0.30, 0.54, 0.90, 0.90). The values for *x*_3 _were generated as random draws from a uniform distribution. In terms of fitting the models, the mechanics are similar to those presented earlier. The model fits are presented in Table 6.

### When logistic regression breaks

The logistic regression fitting procedure can break down, or exhibit lack of convergence. Typically this means that all of the proportions in one of the groups are zero or one; only the former is realistic in the context of SAGE data. This is natural, in that the maximum likelihood point estimate for the group proportion is 0, and inference for *β *involves the fold change to the proportion in the second group, leading to division by zero. When the proportions are this small, the binomial variability dominates the heterogeneity and the values are completely noninformative with respect to the estimation of overdispersion.

We propose a fix that is iterative in nature in that it requires the logistic fitting routine to be run three times. To illustrate this procedure, we will use the data from tag ATTTGAGAAG in Table 2, with the first two tag counts, those from group 1, set to zero.

The first run of the fitting procedure serves to estimate the overdispersion parameter. This fit uses just the groups that have nonzero counts, omitting the problematic group(s). Here, this involves fitting a single proportion to the six libraries in group 2. The fitted proportion is 0.40%, and the overdispersion estimate is 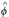 = 3.71*e *- 03.

The second run of the fitting procedure takes the overdispersion parameter as given, and fits the data after replacing the zero proportions in a group with the same small nonzero proportion, giving us a hopefully conservative estimate of the fold change. This type of replacement is commonly used, and is most often justified via the assumption of a vague prior distribution for the proportions, with the point estimate being derived as the posterior mean or mode. A common assumption for a prior in dealing with proportions is the uniform distribution. The posterior mean after 0 successes are observed out of *n*_*i *_trials is 1/(*n*_*i *_+ 1); with multiple trials, it is 1/((∑*n*_*i*_) + 1). This is the value we use. This value is actually quite conservative here, for two reasons. First, the uniform distribution places far too much chance on the possibility of proportions greater than a few percent, which will never be observed with SAGE data. Restricting the distribution to be uniform over the range [0, 0.02] should be more than adequate. Second, the presence of overdispersion means that pooling the samples underemphasizes the evidence of a small proportion being supplied by the zero variance of the observed proportions. While we could pursue a more optimal proportion, we choose in this case to simply use the simplistic bound noted above. Here, as the library sizes in the first group are 49610 and 48479, the proportion is 1/(49610 + 48479) and the faked counts are 0.506 = 49610/(49610 + 48479) and 0.494, respectively. Some reformatted results from this fit are shown in Table 7 (Model 1).

The results for this fit are ridiculously "insignificant". The problem lies in the fact that the use of a *t*-value (a Wald test) relies on the approximate normality of the likelihood function in the vicinity of the maximum, and this shape assumption breaks down severely if the number of counts in one group is small. Tests based on changes in the scaled deviance, corresponding to likelihood ratio tests, are better.

The third run of the fitting procedure fits a simpler submodel, in this case a single proportion for all eight libraries, using the same overdispersion estimate so as to measure the change in deviance. The results of this fit are shown in Table 7 (Model 2). The analysis of deviance test for significance gives


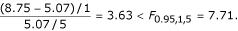


Here, we cannot conclude (given the level of overdispersion) that the difference is real. Note that the degrees of freedom used in the denominator is 5; this follows from the fact that only 6 libraries were used to estimate the overdispersion parameter, and one of those 6 degrees of freedom was needed to estimate the proportion.

In general, when any of the groups has very small counts, checking the change in deviance is a good idea.

## Discussion

Logistic regression with overdispersion addresses three issues with SAGE data: simultaneously modelling multiple types of variance, dealing with multiple groups at once, and allowing for the incorporation of covariates. This procedure is widely implemented in available software. Further, and most importantly, viewing SAGE data in the logistic regression setting supplies the framework for thinking of models that describe such data.

Dealing with multiple types of variance yields significance estimates we believe to be superior to those derived from pooled counts or from *t*-tests. The regression setting carries with it other benefits, such as a well-developed body of work regarding model checking, residual analysis, and detection of outliers. For example, the influence of any given library tag count on the overall analysis can be assessed, and methods can be made more robust by bounding these functions so that no single library drives the results.

There are some areas in which we can identify difficulties and see room for improvement.

First, the model that we are using for the error may be improved. For SAGE data, the proportion associated with a specific tag is rarely on the order of a percent, so

logit(*p*_*i*_) ≈ log(*p*_*i*_)

and we can speak of working with the log rather than the logit transform if we prefer. Assuming variance stability on the log scale then leads to the lognormal distribution often assumed in dealing with microarray data. Assuming a lognormal distribution is equivalent to introducing overdispersion in yet another way, namely as a random effect acting on the *β *scale. Here, the true proportion for library *i *is of the form

logit(*p*_*i*_) = *β*_0 _+ *β*_1_*x*_*i *_+ *ε*_*i*_,

where *ε*_*i *_is a normal random variable with mean 0 and variance 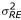. The model described here is a special case of a generalized linear mixed model (GLMM), where "mixed" refers to the fact that we have both fixed effects of interest, the changes with the covariates, and random shocks whose variance needs to be estimated and allowed for. Williams [21] suggests how this model might be fit using a Taylor-series type expansion, again invoking IRLS. However, as noted in Collett [18], p.272, "This approach is not entirely satisfactory for fitting such models to binary data, since the estimates can be biased in certain circumstances. Moreover, the deviance calculated for such models is often very approximate and cannot be recommended for general use in comparing alternative models." There are maximum-likelihood based approaches for fitting GLMMs available in SAS and S-PLUS, but there are known problems with fitting mixed effects models to binary data with small numbers of clusters or libraries. One way of addressing this issue more precisely is via simulation (for example via BUGS [22]). We are exploring these different error models now.

Second, the approach developed above works on one tag at a time. In doing so, it is not exploiting to the fullest the unique features of SAGE data. Examples of such exploitation include correcting for sequencing errors by looking at neighbors where sequence similarity is used to define a neighborhood network, and borrowing strength across genes by using common estimation of parameters such as *φ *over like groups. Work on these issues is ongoing (eg, Colinge and Feger [23], N. Blades (2002), unpublished dissertation, Johns Hopkins) and we think these features could be usefully combined with the approach presented here.

## Methods

### Data

The data used here were initially described in Zhang et al. [2]. The actual numerical libraries used were downloaded from the **SAGE Genie **web resource introduced by Boon et al. [24,25]. These libraries have had the linker tags removed.

### Overdispersed logistic regression

Only a cursory description of the approach is given here; more detailed treatments are given in Collett [18] and McCullagh and Nelder [19], among others.

We want to fit the observed proportions, *p*_*i *_= *Y*_*i*_/*n*_*i*_, as a function of the covariates *X*_*i*_. The first step in this process is to specify what form the relationship will take. If the relationship is linear, so that *p*_*i *_= *β*_0 _+ *β*_1_*X*_*i *_+ *ε*, then we can potentially get fitted proportions outside of the interval [0,1], so we typically choose to fit a transformed version of the *p*_*i*_s as being linear in the covariates. A common choice for proportions is the logistic transformation, *logit*(*p*_*i*_) = *log*(*p*_*i*_/1 - *p*_*i*_) = *β*_0 _+ *β*_1_*X*_*i *_+ *ε*. This particular choice is suggested by the form of the likelihood function for binomial data (see McCullagh and Nelder [19], p.28–32), and we shall take it as assumed here, save to note that while the logit can range over all real values, the corresponding proportions are all between 0 and 1. At this point we are fitting a straight line to a transformed version of the data; this is akin to standard linear regression which is fit by minimizing the sum of squared deviations between the observations and their fitted values: the method of least squares. Now, the default assumption in least squares is that all of the observations are known with equal precision, and hence receive equal weight. This is not the case here, as the variance of a proportion is *V*(*p*_*i*_) = *p*_*i*_(1 - *p*_*i*_)/*n*_*i*_, so that the precision with which an observation is known depends on both the value of that observation and on the size of the total *n*_*i *_from which the proportion was derived. In the case where the observations are known with differing precisions, then the standard adjustment is to fit a weighted version of least squares, minimizing a weighted sum of the squared differences between the observations and their fitted values, where the weights are inversely proportional to the variances of the observations. Thus, at the first step we fit a logistic curve using weighted least squares where the weights are inversely proportional to the variances associated with our initial estimates of the proportions, (*Y*_*i *_+ 0.5)/(*n*_*i *_+ 1). After this first fit, we now have predicted values for each of the observations, and these predicted values in turn suggest new values for the variances and hence the weights. Thus, the second step is to refit the data using the new weights. This process is iterated (iteratively reweighted least squares, IRLS) until the changes in the predicted values from one fit to the next are small enough that the procedure is said to have converged.

Even after the process has converged, it is often the case that the sizes of the squared deviations will be substantially larger than might be expected if the variances were of exactly the form given above. In this case, the data are said to exhibit overdispersion relative to the postulated model, and we seek to estimate the scale of the overdispersion. We deal with the quasi-likelihood case of overdispersion here, where the variance is really of the form *V*(*p*_*i*_) = *n*_*i*_*p*_*i*_(l - *p*_*i*_)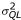, for 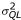 > 1. The added mechanics for computing the hierarchical form are somewhat involved and we refer the reader to Williams [21] for details. Using the quasi-likelihood model for overdispersion, the actual parameters of the best fitting model will not change, as the weights used in the weighted least squares routine are all proportional to the inverses of the variances, and scaling all of the variances by the same factor leaves the relative sizes of the weights unchanged. What does change is the presumed precision associated with these parameters; the variances of the parameters will likewise be multiplied by 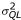, and significance tests need to be adjusted accordingly. In order to estimate 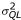, we return to the weighted squared deviations between observations and predictions noted above. Ideally, the sum of the squared weighted residuals will have a chi-squared distribution with *k *- *p *degrees of freedom, where *k *is the total number of libraries and *p *is the number of *β *terms being estimated. As the mean of a chi-squared distribution is equal to its degrees of freedom, we get our initial estimate of 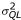 by dividing the sum of squared weighted residuals by the posited degrees of freedom:


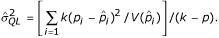


Given the estimated value of 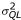, the test statistics are scaled by 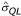 and the significances recomputed. In the cases below, we outline the procedure and couple the descriptions with scripts for the freeware package *R*. In each case, the approach begins by loading the data corresponding to the tag counts *Y*_*i *_and the library sizes *n*_*i*_, which are used to supply the observed proportions. The main distinction between the cases resides in how the covariate *X *values are defined. All of the models assume the presence of a constant vector *X*_0 _of all ones; this produces the corresponding estimate for *β*_0_. Our discussion will likewise treat this covariate as present in all modelling steps.

### Annotated R code

# Source code for models used in the paper

# "Overdispersed Logistic Regression for

# SAGE: Modelling Multiple Groups and

# Covariates", by Baggerly et al.

##########################################

# First, we deal with the case of two

# groups, and introduce the methods for

# fitting the logistic regression models.

##########################################

if(0){

# Load the tag counts for ATTTGAGAAG (y)

# from the 8 libraries in Zhang et al.

# [2], the associated library sizes (n)

# and the covariate vector indicating

# which of two groups the librares

# belong to, normal or cancer (x).

y <- c(320, 600, 312, 549,

246, 65, 41, 52);

n <- c(49610, 48479, 41371, 55700,

60682, 55641, 51294, 61148);

x <- c(0, 0, 1, 1, 1, 1, 1, 1);

# Now fit a standard logistic regression

# model to the data, with no allowance

# for overdispersion. This is done

# through a call to the generalized

# linear model (glm) routine. help(glm)

# provides more information about the

# nature of the arguments here.

fit1 <- glm(cbind(y, n-y) ~x,

family=binomial);

# check the results

summary(fit1);

# Next, we refit the model while

# allowing for overdispersion of the

# quasilikelihood type; all variances

# are inflated by a common factor. This

# call differs from the first only in

# the definition of the glm "family" to

# be used.

fit2 <- glm(cbind(y, n-y) ~x,

family=quasibinomial);

# check the results

summary(fit2);

# Ideally, the sum of the squared

# Pearson residuals should have a chi-

# squared distribution, with mean equal

# to its degrees of freedom. Dividing

# the sum by the degrees of freedom

# gives our initial estimate of the

# overdispersion parameter.

varQL <- sum(residuals(fit2,

"pearson")^2)/fit2$df.residual;

# Finally, we refit the model using the

# overdispersion method suggested by

# Williams [21], where the variances are

# inflated by factors that are slightly

# different depending on the underlying

# library sizes. This routine is

# implemented in the R package "dispmod"

# which is available at

# 

library("dispmod");

fit3 <- glm.binomial.disp(fit1);

# check the results

summary(fit3);

phi <- fit3$dispersion;

# Note that the reported p-values from

# this fit are incorrect. This is due to

# the assumption that the test-stats

# have normal distributions, even though

# we have had to estimate the

# overdispersion parameter. When we have

# to perform this estimation, the

# correct test is a t-test, with a

# number of degrees of freedom

# corresponding to the number of

# libraries less the number of estimated

# parameters. As the number of libraries

# is typically not large, this can

# create a large difference.

sumfit3 <- summary(fit3);

t.values <- summary(

fit3)$coefficients [,"z value"];

p.values <- 2 * pt(-abs(t.values),

fit3$df.residual);

}

##########################################

# Next, we deal with three groups

##########################################

if(0){

# We begin by focusing on gains

# available when multiple groups are

# present, even if the other groups are

# not directly part of the contrast of

# interest, due to the additional

# information that the added groups can

# provide about the scale of the

# overdispersion.

# Here, we use the data from the tag

# TGCTGCCTGT, and this time we note that

# there are 3 groups of libraries:

# normals (libraries 1–2), primary

# tumors (libraries 3–4), and cell lines

# (libraries 5–8). If we are interested

# in the contrast between normals and

# primary tumors, we can fit this using

# only the data from those two groups,

# or using the data from all three.

# First, fit the model as if there were

# just two groups present.

y <- c(0, 1, 1, 15);

n <- c(49610, 48479, 41371, 55700);

x <- c(0, 0, 1, 1);

fit1 <- glm(cbind(y, n-y) ~x,

family=binomial);

fit2 <- glm.binomial.disp(fit1);

# get the correct p-values

fit2.t.values <- summary(

fit2)$coefficients [,"z value"];

fit2.p.values <- 2 * pt(-abs(

fit2.t.values), fit2$df.residual);

# Next, fit the model assuming that

# there are three groups. In this case,

# we cannot use a single covariate

# vector x, as this is not suited to

# indicating 3 or more groups in an

# unordered fashion (using 0, 1, and 2

# for the three groups respectively

# would force an ordering by saying that

# primary tumors are intermediate

# betwixt normal samples and cell lines)

# In general, if we have k groups, we

# need to use k-1 covariate vectors.

# Here, we use

# x1 <- c(0, 0, 1, 1, 0, 0, 0, 0);

# x2 <- c(0, 0, 0, 0, 1, 1, 1, 1);

# The set of all 0s (x1 = 0, x2 = 0)

# corresponds to the first group, here

# the normals, and the other groups are

# defined by which one of the other

# covariates is nonzero:

# Group 2 (primaries), (x1 = 1, x2 = 0),

# Group 3 (cell lines), (x1 = 0, x2 = 1)

y <- c(0, 1, 1, 15, 9, 1, 12, 27);

n <- c(49610, 48479, 41371, 55700,

60682, 55641, 51294, 61148);

x1 <- c(0, 0, 1, 1, 0, 0, 0, 0);

x2 <- c(0, 0, 0, 0, 1, 1, 1, 1);

fit3 <- glm(cbind(y, n-y) ~x1 + x2,

family=binomial);

fit4 <- glm.binomial.disp(fit3);

# get the correct p-values

fit4.t.values <- summary(

fit4)$coefficients [,"z value"];

fit4.p.values <- 2*pt(-abs(

fit4.t.values), fit4$df.residual);

# The above approach has fit the model

# with all of the covariates available,

# but in order to perform an analysis of

# deviance we want to fit various

# submodels using the same estimate of

# overdispersion as found here. In this

# case, there are 3 submodels:

fit5 <- glm(cbind(y, n-y) ~x1,

family=binomial,

weights = fit4$disp.weights);

fit6 <- glm(cbind(y, n-y) ~x2,

family=binomial,

weights = fit4$disp.weights);

fit7 <- glm(cbind(y, n-y) ~1,

family=binomial,

weights = fit4$disp.weights);

# alternatively, the anova function can

# be used, but this only considers the

# submodels obtained by adding terms

# sequentially. Thus, we get the

# deviances for beta_0 (the null model),

# beta_0 + beta_1 (adding the x1

# covariate only), and beta_0 + beta_1 +

# beta_2 (adding the x2 covariate to

# what we already have.

fit4.anodev <- anova(fit4);

}

##########################################

# Next, we deal with the case of other

# covariates, possibly continuous.

##########################################

if(0){

# Here, we are using the counts from the

# GCGAAACCCT tag, but we are treating

# the 8 libraries as coming from tissue

# type 1 (libraries 1–4) and tissue type

# 2 (libraries 5–8), with normal tissue

# of both types (libraries 1–2, 5–6) and

# primary tumor of both types (libraries

# 3–4, 7–8). In this hypothetical

# example, we are able to partition the

# changes into effects associated with

# normal/primary differences (x1) or

# tissue 1/tissue 2 differences (x2).

y <- c(167, 566, 64, 98, 33, 47, 40, 27);

n <- c(49610, 48479, 41371, 55700,

60682, 55641, 51294, 61148);

x1 <- c(0, 0, 1, 1, 0, 0, 1, 1);

x2 <- c(0, 0, 0, 0, 1, 1, 1, 1);

fit1 <- glm(cbind(y, n-y) ~x1 + x2,

family=binomial);

fit2 <- glm.binomial.disp(fit1);

# get the correct p-values

fit2.t.values <- summary(

fit2)$coefficients [,"z value"];

fit2.p.values <- 2*pt(-abs(

fit2.t.values), fit2$df.residual);

# Next, again using the tag as above, we

# posit that we also have access to the

# levels of a biomarker potentially

# predictive of survival, supplied as

# the levels of another covariate x3.

# The values supplied here were

# generated as random draws from a

# uniform (0,1) distribution

x3 <- c(0.89, 0.35, 0.66, 0.23,

0.30, 0.54, 0.90, 0.90);

fit3 <- glm(cbind(y, n-y) ~x1 + x2 + x3,

family=binomial);

fit4 <- glm.binomial.disp(fit3);

# get the correct p-values

fit4.t.values <- summary(

fit4)$coefficients [,"z value"];

fit4.p.values <- 2*pt(-abs(

fit4.t.values), fit2$df.residual);

}

## Authors' contributions

KAB, LD and JSM developed the main ideas and the methodology; LD did most of the coding. CMA supplied SAGE data and provided practical feedback on aspects of earlier approaches found to be wanting, thus guiding further development.

## Figures and Tables

**Table 1:** Methods of summarizing data. The effects of pooling and reduction to proportions on a single tag measured across four libraries. Pooling reduces the data to the summed counts at the right, and focusing on proportions reduces the data to the proportions on the bottom. In both cases, information is lost.

					Summed Counts
Tag Count	*Y*_1_	*Y*_2_	*Y*_3_	*Y*_4_	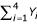
Library Size	*n*_1_	*n*_2_	*n*_3_	*n*_4_	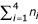
Proportions	*Y*_1_/*n*_1_	*Y*_2_/*n*_2_	*Y*_3_/*n*_3_	*Y*_4_/*n*_4_	

**Table 2:** Tag counts from sample SAGE libraries. Counts and proportions of tags ATTTGAGAAG, TGCTGCCTGT and GCGAAACCCT in 8 colon libraries from Zhang et al. [2]; two normal colon (NC), two primary tumors (TU) and four cell lines.

Group	Normal Colon		Primary Tumor		Cell Lines			
Library	NC1	NC2	TU98	TU102	CACO2	HCT116	RKO	SW837

ATTTGAGAAG	320	600	312	549	246	65	41	52
TGCTGCCTGT	0	1	1	15	9	1	12	27
GCGAAACCCT	167	566	64	98	33	47	40	27
Library Size	49610	48479	41371	55700	60682	55641	51294	61148
Propn ATT..(%)	0.65	1.24	0.75	0.99	0.41	0.12	0.08	0.09

**Table 3:** Logistic regression models for two groups. Logistic regression fits contrasting normal colon with cancer samples for tag ATTTGAGAAG from Table 2. The first model makes no allowance for overdispersion, and the latter two introduce it in different ways. The introduction of overdispersion is important as it dramatically affects the results, but the choice of overdispersion method is less crucial.

Model 1:	No overdispersion	*V*(*Y*_*i*_) = *n*_*i*_*p*_*i*_(1 - *p*_*i*_)		
Coefficients	Estimate	(s.e)	z-value	p-value
*β*_0_	-4.660	0.033	-140.68	< 2*e*-16
*β*_1_	-0.888	0.043	-20.41	< 2*e*-16

Model 2:	Quasilikelihood	*V*(*Y*_*i*_) = *n*_*i*_*p*_*i*_(1 - *p*_*i*_) 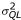		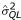 = 187.6

Coefficients	Estimate	(s.e)	t-value	p-value
*β*_0_	-4.660	0.454	-10.261	5*e *- 05
*β*_1_	-0.888	0.595	-1.489	0.187

Model 3:	Hierarchical	*V*(*Y*_*i*_) = *n*_*i*_*p*_*i*_(1 - *p*_*i*_) [1 + (*n*_*i *_- 1)*φ*]		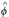 = 3.4*e *- 03

Coefficients	Estimate	(s.e)	t-value	p-value
*β*_0_	-4.656	0.428	-10.874	3.6*e *- 05
*β*_1_	-0.850	0.570	-1.492	0.186

**Table 4:** Expanding contrasts from two to three groups. Logistic regression models testing the significance of a difference between normal colon and primary tumor (*β*_1_) for tag TGCTGCCTGT from Table 2. In the first model, only data from the four libraries directly involved are used. In the second model, data from the four cell line libraries are also included, providing a more stable estimate of the overdispersion parameter *φ*.

Model 1:	Two Groups	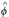 = 8.938*e *- 05		*df *= 2
Coefficients	Estimate	(s.e)	t-value	p-value
*β*_0_	-11.484	2.309	-4.973	0.038
*β*_1_	2.681	2.388	1.123	0.378

Model 2:	Three Groups	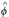 = 1.160*e *- 04		*df *= 5

Coefficients	Estimate	(s.e)	t-value	p-value
*β*_0_	-11.484	2.574	-4.462	0.007
*β*_1_	2.676	2.661	1.005	0.361
*β*_2_	3.020	2.604	1.159	0.299

**Table 5:** Analysis of deviance. Deviance table for various submodels fit to the data for tag TGCTGCCTGT given in Table 2. All of these models use the value for overdispersion found for the most extensive model, 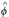 = 1.160*e *- 04.

Terms Fitted	Deviance	d.f.
*β*_0_	9.7433	7
*β*_0 _+ *β*_1_	9.7418	6
*β*_0 _+ *β*_2_	7.9826	6
*β*_0 _+ *β*_1 _+ *β*_2_	5.7866	5

**Table 6:** Incorporating covariates into the model. Models treating the fitting of counts for tag GCGAAACCCT from Table 2, with the cell lines hypothetically allocated to normal tissue B (libraries 5 and 6) and cancer tissue B (libraries 7 and 8). This division is made to illustrate how the effects of two differences, normal vs cancer and tissue A vs tissue B (*β*_1 _and *β*_2 _respectively) can be partitioned according to their importance. In Model 2, we have further introduced a continuous covariate (*β*_3_) corresponding to the levels of a biomarker to show how that can be figured in as well.

Model 1:	Hypothetical Cov.	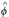 = 1.224*e *- 03		df = 5
Coefficients	Estimate	(s.e)	t-value	p-value
*β*_0_	-4.928	0.291	-16.921	1.318e - 05
*β*_1_	-1.293	0.593	-2.181	0.0810
*β*_2_	-1.956	0.738	-2.650	0.0454

Model 2:	Hypothetical Biom.	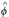 = 1.254*e *- 03		df = 4

Coefficients	Estimate	(s.e)	t-value	p-value
*β*_0_	-4.167	0.608	-6.851	1.012e-03
*β*_1_	-1.423	0.611	-2.328	0.0674
*β*_2_	-2.031	0.752	-2.700	0.0428
*β*_3_	-1.365	1.028	-1.328	0.2417

**Table 7:** Fitting nested deviance models. Fitting nested models to the data in order to get deviance scores. The difference in deviance between models is a better indicator of the significance of the associated effect (*β*_1_) when the logistic regression fits are near the boundary of the space, giving proportions close to zero.

Model 1:	Full Model	Deviance = 5.0742		
Coefficients	Estimate	(s.e)	t-value	p-value
*β*_0_	-11.494	13.518	-0.06	0.9519
*β*_1_	5.987	13.524	-0.03	0.9750

Model 2:	Null Model	Deviance = 8.7541		

Coefficients	Estimate	(s.e)	t-value	p-value
*β*_0_	-5.794	0.392	-14.772	6.05e-06

## References

[B1] Velculescu VE, Zhang L, Vogelstein B, Kinzler KW (1995). Serial analysis of gene expression. Science.

[B2] Zhang L, Zhou W, Velculescu VE, Kern SE, Hruban RH, Hamilton SR, Vogelstein B, Kinzler KW (1997). Gene expression profiles in normal and cancer cells. Science.

[B3] Madden SL, Galella EA, Zhu J, Bertelsen AH, Beaudry GA (1997). SAGE transcript profiles for p53-dependent growth regulation. Oncogene.

[B4] Audic S, Claverie JM (1997). The significance of digital gene expression profiles. Genome Res.

[B5] Kal AJ, van Zonneveld AJ, Benes V, van den Berg M, Koerkamp MG, Albermann K, Strack N, Ruijter JM, Richter A, Dujon B, Ansorge W, Tabak HF (1999). Dynamics of gene expression revealed by comparison of serial analysis of gene expression transcript profiles from yeast grown on two different carbon sources. Mol Biol Cell.

[B6] Chen H, Centola M, Altschul SF, Metzger H (1998). Characterization of gene expression in resting and activated mast cells. J Exp Med.

[B7] Lai A, Lash AE, Altschul SF, Velculescu V, Zhang L, McLendon RE, Marra MA, Prange C, Morin PJ, Polyak K, Papadopoulos N, Vogelstein B, Kinzler KW, Strausberg RL, Riggins GJ (1999). A public database for gene expression in human cancers. Cancer Res.

[B8] Michiels EMC, Oussoren E, van Groenigen M, Pauws E, Bossuyt PMM, Voute PA, Baas F (1999). Genes differentially expressed in medulloblastoma and fetal brain. Physiol Genomics.

[B9] Man MZ, Wang X, Wang Y (2000). POWER_SAGE: comparing statistical tests for SAGE experiments. Bioinformatics.

[B10] Ruijter JM, van Kampen AHC, Baas F (2002). Statistical evaluation of SAGE libraries: Consequences for experimental design. Physiol Genomics.

[B11] Ryu B, Jones J, Blades NJ, Parmigiani G, Hollingsworth MA, Hruban RH, Kern SE (2002). Relationships and differentially expressed genes among pancreatic cancers examined by large-scale serial analysis of gene expression. Cancer Res.

[B12] Baggerly KA, Deng L, Morris JS, Aldaz CM (2003). Differential expression in SAGE: Accounting for normal between-library variation. Bioinformatics.

[B13] Porter DA, Krop IE, Nasser S, Sgroi D, Kaelin CM, Marks JR, Riggins G, Polyak K (2001). A SAGE (serial analysis of gene expression) view of breast tumor progression. Cancer Res.

[B14] Ryu B, Jones J, Hollingsworth MA, Hruban RH, Kern SE (2001). Invasion-specific genes in malignancy: Serial analysis of gene expression comparisons of primary and passaged cancers. Cancer Res.

[B15] Nacht M, Dracheva T, Gao Y, Fujii T, Chen Y, Player A, Akmaev V, Cook B, Dufault M, Zhang M, Zhang W, Guo M, Curran J, Han S, Sidransky D, Buetow K, Madden SL, Jen J (2001). Molecular characteristics of non-small cell lung cancer. Proc Nat Acad Sci USA.

[B16] Greller LD, Tobin FL (1999). Detecting selective expression of genes and proteins. Genome Res.

[B17] Stekel DJ, Git Y, Falciani F (2000). The comparison of gene expression from multiple cDNA libraries. Genome Res.

[B18] Collett D (2002). Modelling Binary Data, 2e.

[B19] McCullagh P, Nelder JA (1989). Generalized Linear Models, 2e.

[B20] Crowder MJ (1978). Beta-binomial ANOVA for proportions. Appl Stat.

[B21] Williams DA (1982). Extra-binomial variation in logistic linear models. Appl Stat.

[B22] Best NG, Spiegelhalter DJ, Thomas A, Brayne CEG (1996). Bayesian analysis of realistically complex models. J Royal Stat Soc A.

[B23] Colinge J, Feger G (2001). Detecting the impact of sequencing errors on SAGE Data. Bioinformatics.

[B24] Boon K, Osorio EC, Greenhut SF, Schaefer CF, Shoe maker J, Polyak K, Morin PJ, Buetow KH, Strausberg RL, de Souza SJ, Riggins GJ (2002). An anatomy of normal and malignant gene expression. Proc Nat Acad Sci USA.

[B25] SAGE Genie. http://cgap.nci.nih.gov/SAGE.

